# Suicide Ideation, Plans, and Attempts Attributed to the COVID-19 Pandemic Among US Veterans

**DOI:** 10.1001/jamanetworkopen.2023.20193

**Published:** 2023-06-26

**Authors:** Ian H. Stanley, Kathleen M. Flarity, Michael D. April

**Affiliations:** 1 Center for COMBAT Research, Department of Emergency Medicine, University of Colorado School of Medicine, Aurora; 2 Department of Emergency Medicine, School of Medicine, University of Colorado Anschutz Medical Campus, Aurora; 3 Department of Military and Emergency Medicine, Uniformed Services University, Bethesda, Maryland

## Abstract

This cross-sectional study analyzes data from the 2021 National Survey on Drug Use and Health to assess whether suicidal experiences among US veterans are associated with the COVID-19 pandemic.

## Introduction

Over the past 2 decades, the age- and sex-adjusted suicide rate has been higher for US veterans compared with nonveterans.^1^ The COVID-19 pandemic spurred concerns about increased suicide risk, including among veterans.^2^ Nevertheless, in 2019 and 2020, the US experienced population-level declines in the suicide rate,^3^ and declines were greater among veterans (9.7%) than nonveteran adults (5.5%).^1^ Veterans may have experienced a differential effect of the COVID-19 pandemic on suicide rates or proxy indicators, such as suicide ideation, plans, and attempts.

## Methods

In this cross-sectional study, we analyzed data from the 2021 National Survey on Drug Use and Health (NSDUH), a representative survey of noninstitutionalized US civilians 12 years and older. Race and ethnicity data were not analyzed for this study, and participants provided informed consent to the NSDUH. Data were collected from January 14 to December 20, 2021. The NSDUH excluded current active duty military personnel. Additional information on the NSDUH survey methods has been published elsewhere.^4^ The Colorado Multiple Institutional Review Board approved the study protocol, and we followed the STROBE reporting guideline.

We analyzed responses from adults 18 years and older. We determined veteran status by affirmative responses to the question, “Have you ever been in the United States Armed Forces?” Participants were queried if, during the past 12 months, they had serious thoughts about suicide (yes or no). Those who responded yes were then asked if, during the past 12 months, they made a suicide plan or suicide attempt (yes or no). After each question assessing past-year suicidality, participants were asked, “Was this because of the COVID-19 pandemic?” (yes or no). Additionally, we assessed responses to the analog question, “Since the beginning of the COVID-19 pandemic, how much, if at all, has COVID-19 negatively affected your emotional or mental health?” (not at all, a little, some, quite a bit, or a lot). We used logistic regression analyses examining the association between veteran status (1 indicates veteran; 0, nonveteran) and COVID-19–related suicidality (1, yes; 0, no) or adverse mental health (1, quite a bit or a lot; 0, not at all, a little, or some), controlling for age and sex. We used NSDUH-calculated sampling weights in analyses.^4^ We considered 2-sided *P* < .05 statistically significant and used SPSS, version 29.0 (IBM Corp) for statistical analyses.

## Results

This study included 47 291 adults 18 years and older (48.6% men, 51.4% women, and 7.8% veterans) (Table 1). Overall, 4.8% reported suicide ideation; 1.3%, suicide plans; and 0.7%, suicide attempts in the past year. Veterans were at increased odds of reporting report past-year suicide ideation (adjusted odds ratio [AOR], 1.42 [95% CI, 1.41-1.42]), suicide plans (AOR, 1.97 [95% CI, 1.96-1.98]), and suicide attempts (AOR, 2.94 [95% CI, 2.92-2.95]) (Table 2). However, among individuals reporting past-year suicidal experiences, veterans had decreased odds of attributing their suicidal experiences to the COVID-19 pandemic compared with nonveterans (suicide ideation: AOR, 0.53 [95% CI, 0.52-053]; suicide plans: AOR, 0.45 [95% CI, 0.44-0.46]; suicide attempts: AOR, 0.39 [95% CI, 0.38-0.39]) (Table 2). Veterans were also at decreased odds of reporting adverse mental health, broadly, related to COVID-19 compared with nonveterans (AOR, 0.89 [95% CI, 0.89-0.89]) (Table 2).

**Table 1.** Participant Characteristics From the 2021 National Survey on Drug Use and Health

Characteristic	Participant group, No. (weighted %)		
	Overall sample	Veterans^a^	Nonveterans
Age, y			
18-25	13 979 (13.2)	195 (1.9)	13 779 (14.1)
26-34	9588 (15.8)	360 (7.6)	9223 (16.5)
35-49	12 561 (24.5)	618 (14.9)	11 940 (25.3)
50-64	5725 (24.8)	515 (25.3)	5206 (24.7)
≥65	5438 (21.7)	1011 (50.3)	4424 (19.3)
Sex			
Men	20 901 (48.6)	2263 (90.2)	18 632 (45.1)
Women	26 390 (51.4)	436 (9.8)	25 940 (54.9)

^a^ Data on veteran status were missing for 20 respondents.

**Table 2.** Logistic Regressions Comparing Past-Year Suicide-Related Experiences Among US Veterans and Nonveterans

Experience	Participant group, No. (weighted %)			Age- and sex-adjusted comparisons of US veterans vs nonveterans	
	Overall sample	Veterans	Nonveterans	AOR (95% CI)	*P* value
Overall suicide-related experiences					
Suicide ideation	3177 (4.8)	154 (3.4)	3022 (4.9)	1.42 (1.41-1.42)	<.001
Suicide plans	972 (1.3)	45 (1.0)	927 (1.3)	1.97 (1.96-1.98)	<.001
Suicide attempts	435 (0.7)	18 (0.7)	417 (0.7)	2.94 (2.92-2.95)	<.001
Suicide-related experiences attributed to COVID-19					
Suicide ideation^a^	540 (15.6)	17 (9.2)	523 (16.0)	0.53 (0.52-0.53)	<.001
Suicide plans^a^	146 (12.7)	6 (7.8)	140 (13.1)	0.45 (0.44-0.46)	<.001
Suicide attempts^a^	79 (14.3)	4 (9.9)	75 (14.6)	0.39 (0.38-0.39)	<.001
Adverse mental health	7876 (14.4)	251 (8.5)	7623 (14.9)	0.89 (0.89-0.89)	<.001

Abbreviation: AOR indicates adjusted odds ratio.

^a^ Among those reporting suicide ideation, suicide plans, or suicide attempts.

## Discussion

There is no single contributing factor to suicidal thoughts and behaviors.^1,5^ However, in this cross-sectional study, US veterans were at 47% to 61% decreased odds compared with nonveterans of attributing their past-year suicidal experiences to the COVID-19 pandemic. Veterans may be resilient to the psychosocial sequelae of tragedies, such as COVID-19,^6^ perhaps due to their military experiences. Nevertheless, our findings suggest that veterans continue to experience disparities in the prevalence of past-year suicidal thoughts and behaviors, underscoring the need for a multilayered suicide prevention approach. A recent report^3^ indicates that following the 2-year decline, suicide rates increased in 2021—the latest year for which data are available—among the general population (2021 data on veterans are not yet published). Study limitations include lack of data on the severity and chronicity of respondents’ suicidality, nuanced information on veteran status, and other potential contributors to respondents’ suicidal thoughts and behaviors.

## Notes

Supplement.
Data Sharing Statement
