# Supplementary material for: Suicide Ideation, Plans, and Attempts Attributed to the COVID-19 Pandemic Among US Veterans
Source: JAMA Netw Open. 2023 Jun 26;6(6):e2320193. doi: 10.1001/jamanetworkopen.2023.20193 (PMC10293906; doi:10.1001/jamanetworkopen.2023.20193)
Supplement: Supplement. — Data Sharing Statement [file jamanetwopen-e2320193-s001.pdf]

Data Sharing Statement

Stanley. Suicidal Ideation, Plans, and Attempts Attributed to the COVID-19 Pandemic Among US Veterans. *JAMA Netw Open*. Published June 26, 2023.
doi:10.1001/jamanetworkopen.2023.20193

Data
Data available: No

Additional Information
Explanation for why data not available: Data are managed by SAMHSA and are available to the public for download (https://www.datafiles.samhsa.gov/dataset/national-survey-drug-use-and-health-2021-nsduh-2021-ds0001).
